# Magnetic Resonance Imaging of Clubfoot Treated With the Ponseti Method: A Short-Term Outcome Study

**DOI:** 10.3389/fped.2022.924028

**Published:** 2022-07-05

**Authors:** Jiangchao Zhang, Ningqing Wang, Haixiang Lv, Zhenjiang Liu

**Affiliations:** Department of Orthopedics, Children’s Hospital, Capital Institute of Pediatrics, Beijing, China

**Keywords:** congenital talipes equinovarus, clubfoot, Ponseti method, magnetic resonance imaging, deformity congenital talipes equinovarus, deformity

## Abstract

**Objective:**

To quantitatively evaluate the effectiveness of the Ponseti method for the correction of clubfoot, we decided to use magnetic resonance imaging (MRI) to evaluate changes in the tarsal bone relationship.

**Methods:**

This is a retrospective study of fifteen children with clubfeet who were treated with the Ponseti method. MRI studies were obtained using a 3.0T Machine (GE Healthcare, United States). T1-weighted and T2-weighted images were acquired in the standard anatomic sagittal, transverse, and coronal planes. For the measurement, the best slice that clearly demonstrated the anatomy was chosen. Sagittal talocalcaneal angle, sagittal tibiocalcaneal angle, coronal tibiocalcaneal angle, transverse talar neck angle, transverse talonavicular angle, and transverse talocalcaneal angle were measured. The eighteen corrected clubfeet were compared with the twelve unilateral normal feet at clinical and radiological levels using a Pirani scoring system and MRI, respectively.

**Results:**

In total, 15 cases (twelve boys and three girls) with clubfeet were examined by using MRI. Twelve cases had unilateral and three had bilateral involvement (eleven left clubfeet and seven right clubfeet), giving a total of eighteen clubfeet when compared with twelve normal feet. The mean age of patients at examination was 47.7 months (8–96 months). The recovery of the corrected clubfoot in these patients met the goals of Ponseti treatment (functional, normal looking, pain-free, and plantigrade foot). Before Ponseti treatment, the mean Pirani score of clubfoot was 5.5 (5–6). During this follow-up, the Pirani score was 0.07 (0–0.05). The results of the MRI indicated that only the transverse talonavicular angle showed a significant difference between the treated clubfeet and the normal feet (*p* < 0.001). One case had dorsal talonavicular subluxation in the sagittal plane and had the lateral subluxation of the navicular in the transverse plane, which has never been reported in previous studies.

**Conclusion:**

Although the appearance and function of clubfoot were recovered well after the Ponseti method, the results of MRI indicated that the Ponseti method successfully corrected the varus, cavus, and equinus deformities and incompletely corrected the adduction deformity regarding transverse talonavicular angle. At the same time, the Ponseti method may cause dorsal talonavicular subluxation in the sagittal plane and lateral subluxation of the navicular in the transverse plane on MRI.

## Introduction

Clubfoot (congenital talipes equinovarus) is a well-known common pediatric foot deformity, with an incidence of 1–2 per 1,000. Affected individuals present unilateral or bilateral clubfoot and involvement of both feet occurs in approximately 50% of cases. It affects men more than women, where the men to women ratio of clubfoot is 2:1. Forefoot adducts, midfoot cavus, hindfoot varus, and ankle equinus are all characteristics of this deformity (1). The traditional treatment is early postnatal non-surgical therapy, such as the Ponseti method, which has been accepted as the gold standard treatment of clubfoot in many countries. The Ponseti method consists of two phases: the treatment phase and the maintenance phase. The treatment phase is involved with manipulation, weekly castings for the period of 6 weeks, and percutaneous Achilles tenotomy (PAT). The maintenance phase is involved with bracing after the casting phase to maintain the corrected clubfoot and prevent the recurrence (2). Ponseti suggested that if the patient had a functional, plantigrade foot with adequate mobility, the result of treatment should be considered successful (3). Derzsi suggested that the Ponseti method in the treatment of clubfoot resulted in satisfactory clinical results; however, there were still abnormal differences in imaging studies (4).

A number of x-ray, computed tomography (CT), and computerized three-dimensional reconstructions on CT, arthrography, infrared thermal imaging, and ultrasound (US) studies on clubfoot have been performed in recent years (5–13). Due to the tarsal bones of infants are not completely ossified and are primarily cartilaginous, it has been difficult to assess the morphology and alignment of tarsal bones using x-ray and CT. Arthrography has been recommended for better visualization of tarsal bones, such as cartilage, but this is an invasive procedure (11). We can see various elements of the deformity by using US, but no quantitative evaluations can be made (9). With magnetic resonance imaging (MRI), we have a unique opportunity to photograph the chondro-osseous components and soft tissue anomalies of clubfoot in multiple planes. The purpose of our study was to use MRI to quantitatively evaluate changes in the tarsal bone relationship of corrected clubfoot so as to objectively describe the effectiveness of Ponseti method.

## Materials and Methods

Clinical and MRI imaging data of children with clubfoot were treated at our pediatric orthopedic clinic between March 2014 and March 2022 and were retrospectively analyzed. Inclusion criteria were as follows: children with clubfeet who were treated with the Ponseti methods, which involve gentle manipulation, weekly castings for the period of 4–6 weeks, PAT, and use of foot abduction orthosis. Exclusion criteria were as follows: (i) incomplete case data; (ii) unsatisfactory MRI imaging, such as improper cross-sectional images of the tarsal bones; and (iii) patients or parents declined to undergo the MRI examinations because of sedation and cost issues. Finally, fifteen cases that had MRI were included in our study and the recovery of the corrected clubfoot in our patients met the goals of Ponseti treatment (functional, normal looking, pain-free, and plantigrade foot). We have not found the recurrence of the clubfoot treated with the Ponseti method during the follow-up. Twelve cases were boys and three were girls. Twelve cases had unilateral and three had bilateral involvement (eleven left clubfeet and seven right clubfeet), giving a total of eighteen clubfeet when compared with twelve normal feet. The mean age at examination was 47.7 months (8–96 months). This study was approved by the life ethics committee of the Capital Institute of Pediatrics, Beijing, China (no. SHERLLM2022015), and written informed consent was obtained from their parents. Patients arrived at the MRI scanner were of sleep-deprived and were sedated by oral administration of 0.5 mg/kg of chloral hydrate. MRI was performed on both feet. We used a 3.0T MRI Scanner (GE Healthcare, United States) with foot and ankle coil that generated both T1-weighted and T2-weighted images with slice thickness of 3–4 mm. Each study was assessed with standard sagittal, coronal, and transverse planes. In general, we chose the best slice that clearly demonstrated the anatomy of the foot. At the same time, we used the Pirani scoring system to document the severity of clubfoot deformities (before and after the Ponseti treatment).

Six major measurement parameters were evaluated, which are as follows: sagittal talocalcaneal angle, sagittal tibiocalcaneal angle, coronal tibiocalcaneal angle, transverse talonavicular angle, transverse talar neck angle, and transverse talocalcaneal angle. Two senior pediatric orthopedic surgeons measured these angles separately to reduce interobserver error. In our investigation, results were discarded if the discrepancy between the measured angles was greater than 3.0°. The real value was then calculated as the average of the angles measured by two observers.

**FIGURE 1:**
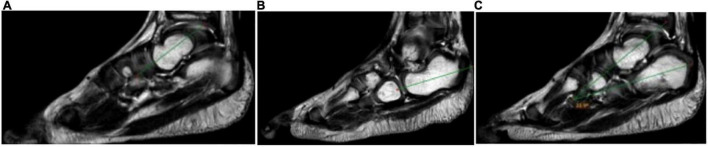
**(A)**: The long axis of talus; **(B)**: the long axis of calcaneus; and **(C)**: sagittal talocalcaneal angle.

**FIGURE 2:**
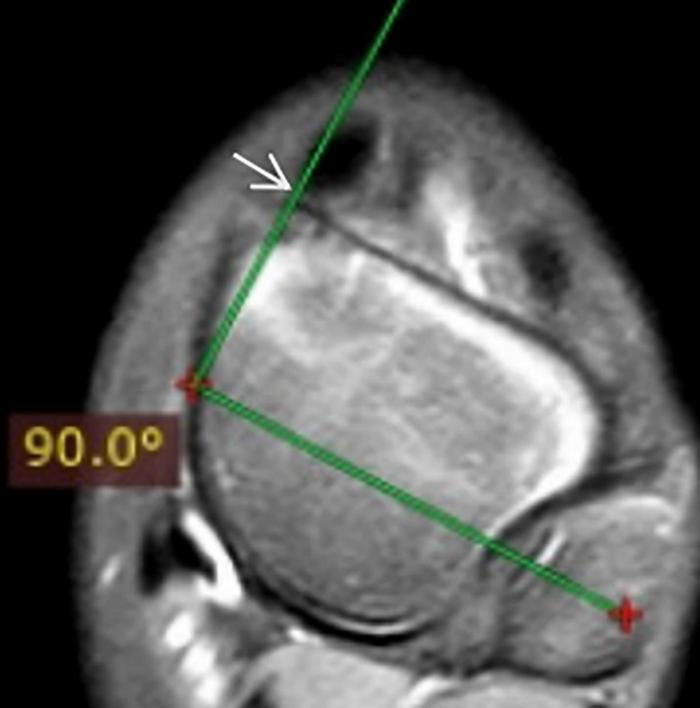
The long axis of the talar body (arrow).

(1) The sagittal talocalcaneal angle was measured by drawing lines through the long axis of the entire ossified and cartilaginous talus and calcaneus bones (Figure 1); (2) the sagittal tibiocalcaneal angle was measured as the angle formed by the long axis of the tibial and calcaneus bones; (3) the coronal tibiocalcaneal angle was defined as the angle formed by the long tibial axis and the line connecting dorsolateral and plantolateral corners of calcaneus; (4) at the transverse plane, the transmalleolar axis was defined as a line bisecting the lateral and medial malleoli, previously described by Jakob (14). A line perpendicular to the transmalleolar axis was accepted as the longitudinal axis of the talar body (Figure 2). The axis of the talar neck was defined by a line passing through the midpoint of the talar head and talar neck. (5) The transverse talar neck angle was considered by drawing lines through the long axis of the talus body and talus head; (6) the transverse talonavicular angle was measured by drawing perpendicular lines to the navicular baseline and to the transmalleolar axis; and (7) the transverse talocalcaneal angle was measured by drawing lines through the long axis of the calcaneus and the talar body. These angles were measured by maintaining a superimposing image.

Continuous data were presented with mean and standard deviation (SD). All statistical analyses were performed using SPSS version 26.0 (IBM, Armonk, NY, United States). Differences between normal feet and treated clubfeet were analyzed using an independent sampled Student’s *t*-test or Mann-Whitney *U* test, as appropriate. *p* < 0.05 was considered statistically significant.

## Results

**FIGURE 3:**
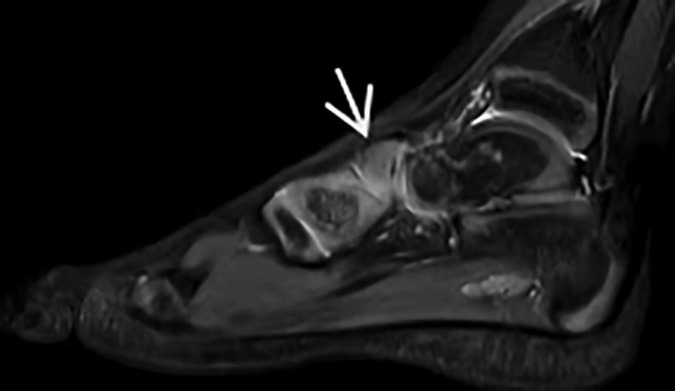
Dorsal talonavicular subluxation in the sagittal plane of MRI (arrow).

**FIGURE 4:**
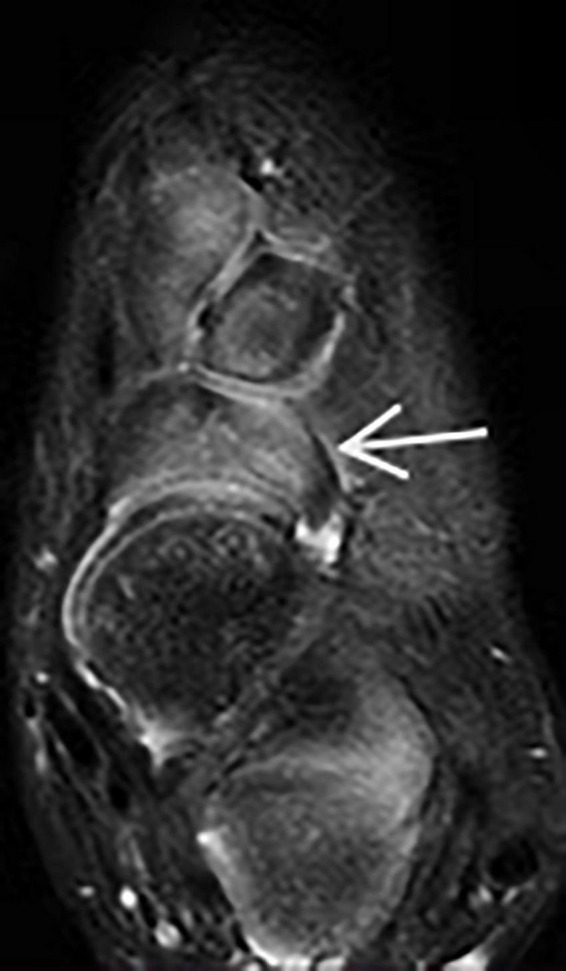
Lateral talonavicular subluxation in the transverse plane of MRI (arrow).

Before the Ponseti treatment, the mean Pirani score of clubfoot was 5.5 (5.0–6.0). The Pirani score at this follow-up period for the corrected clubfoot was 0.07 (range, 0–0.05). Pirani scores showed a statistically significant improvement before starting and after the Ponseti method. Among these major measurement parameters (Table 1), sagittal talocalcaneal angle, sagittal tibiocalcaneal angle, coronal tibiocalcaneal angle, transverse talar neck angle, and transverse talocalcaneal angle showed no significant difference between treated clubfeet and normal feet (*p* > 0.05). The transverse talonavicular angle showed a significant difference between the two groups (*p* < 0.001). We found that one patient had dorsal talonavicular subluxation by MRI (Figure 3). One patient had lateral subluxation of the navicular in the transverse plan on MRI (Figure 4), which has never been described.

**TABLE 1:** Six measurement parameters between clubfeet and normal feet.

	Sagittal tibiocalcaneal angle	Sagittal talocalcaneal angle	Coronal tibiocalcaneal angle	Transverse talonavicular angle	Transverse talar neck angle	Transverse talocalcaneal angle
Clubfoot	73.3 ± 6.6	28.7 ± 7.6	12.7 ± 5.4	45.2 ± 13.3	32.5 ± 6.9	11.3 ± 4.0
Normal	70.5 ± 8.3	29.2 ± 8.4	11.2 ± 2.8	23.5 ± 11.5	26.7 ± 12.1	8.7 ± 4.4
*P-*value	0.320	0.887	0.376	0.001	0.104	0.116

*Data presentation: mean ± standard deviation (SD).*

## Discussion

Clubfoot is a three-dimensional malformation of leg, ankle, and foot that is immediately visible at birth. It is characterized by forefoot adducts, midfoot cavus, hindfoot varus, and equinus of the ankle. To assess the severity of the abnormalities, several pediatric orthopedists used clinical-functional scores (such as the Pirani scores or the Dimeligo scores) and radiological data. Despite the widespread use of analytical radiography in clubfoot, Surendra et al. concluded that radiographic assessment of clubfoot was not a trustworthy tool due to significant intraobserver and interobserver variability (15). Conventional radiographs were radioactive, unreliable, not easily reproducible, and imprecise in assessing and classifying the severity of clubfoot. Meanwhile, because the tarsal bones of these patients are not totally ossified and are primarily cartilaginous, we cannot utilize an x-ray to assess the relationship between the tarsal bones.

Clubfoot with good cosmetic and functional healing, according to Blakeslee, may have several covert tarsal joint impingements, dislocations, or subluxations that are not apparent on clinical examination or radiographs (16). Furthermore, x-ray has not made visualization of the talonavicular relationships possible. With the long-term follow-up of Ponseti-treated clubfoot cases, the investigators found an increase in the recurrence rate of clubfoot, which was ranged from 1.9 to 45% (17). Ponseti suggested that the recurrence of clubfoot may be due to inadequate repositioning of the tarsal bone alignment, which was not diagnosed with clinical or radiological examinations in early childhood. The level of correction can be seen clearly on MRI, which can also reveal complications and relapses before skeletal maturity. Although there have been several reports on MRI findings of tarsal bone abnormalities of clubfoot, MRI studies on the level of correction after the Ponseti method are scarce and have not been reported in China (18–24). Therefore, we decided to objectively evaluate the effectiveness of the Ponseti method for the correction of clubfoot using MRI.

To describe the equinus deformity of clubfoot, the sagittal talocalcaneal angle and sagittal tibiocalcaneal angle were measured in this study. Before Ponseti treatment, the sagittal talocalcaneal angle was reported to be 28 ± 6° in normal foot and 5 ± 9° in clubfoot on MRI by Downey (*p* < 0.05) (18). The mean sagittal talocalcaneal angle in our study was 28.7° in treated clubfoot and 29.2° in the normal foot (*p* > 0.05). The mean sagittal tibiocalcaneal angle was 73.3° in the corrected clubfoot and 70.5° in the normal foot (*p* > 0.05). The results of our MRI revealed that the Ponseti method was successful enough in the correction of equinus deformity of the clubfoot. Pekindil reported the mean sagittal talocalcaneal angle of 36.0° on the normal foot and 31.4° on the treated side (*p* > 0.05) (19). Amhad also reported the mean sagittal tibiocalcaneal angle of 80.2° in the normal side and 91.6° on the corrected side (*p* > 0.05) (22). These measurements were consistent with the findings of our study. An MRI protocol was devised to illustrate the tarsal bone changes that occur with the Ponseti method of the treatment by Pirani, though these changes were qualitative rather than quantitative (21). They discovered that the Ponseti method corrected not only the aberrant relationships of the tarsal bones but also the abnormal shapes of the individual tarsal osteochondral anlages.

The coronal tibiocalcaneal angle was used to assess the varus deformity of the clubfoot. Satio found that the coronal tibiocalcaneal angle was 0 ± 13.8° in the clubfoot before treatment and 14 ± 4.6° in the normal foot (*p* < 0.001) (23). Our results of coronal tibiocalcaneal angle were for normal and corrected foot were statistically insignificant. We believed that the Ponseti method successfully corrected the varus deformity of the clubfoot. Pirani et al. also observed that the abnormal relationship between the calcaneus and tibia of clubfoot had returned to normal in the coronal plane during the third cast fixation phase.

When compared to normal children, the onset of navicular ossification was found to be delayed in children with clubfoot, and the navicular bone was not apparent on radiographs until they were 3–5 years old (25). However, it was easy to see the navicular cartilage in the sagittal plane of MRI. If a substantial cavus deformity cannot be treated by stretching the plantar fascia, Carroll believed that extrusion of the dorsolateral navicular bone will occur, leading to talonavicular subluxation (26). In our study, 1 of 18 (5.6%) of the corrected clubfeet had dorsal talonavicular subluxation. Its rate has been reported as 25% by Ahmad (22). In the MRI transverse plane, we found that 1 corrected clubfoot had lateral talonavicular subluxation, which has never been reported in previous studies. We speculated that the navicular bone had developed from medial displacement to lateral subluxation due to overcorrection of the Ponseti method and cast fixation. MRI can identify these insidious complications much earlier than x-ray.

Adducts deformities of clubfoot-related measurements were transverse talonavicular angle, transverse talar neck angle, and transverse talocalcaneal angle. To our knowledge, it was difficult to identify the longitudinal axis of the talar body in the transverse plane of MRI. The longitudinal axis of the talar body was defined as a line perpendicular to the transmalleolar line passing through the center of the medial and lateral malleoli (14). Before treatment, Downey reported that the mean transverse talar neck angle was 44.0° for clubfoot and 30.8° for normal foot (*p* < 0.01) (18). In addition, the mean transverse talocalcaneal angle was 22.8° in clubfoot vs. 10.1° in the normal foot (*p* < 0.05). These findings were consistent with the adducts deformity of clubfoot described by Ponseti et al. In our study, only transverse talonavicular angle of these three measurements for normal foot and corrected clubfoot were statistically significant. Kamegaya performed plaster fixation on children with clubfoot, they reported 21.0 ± 9.5° for normal foot and 44.2 ± 15.9° for treated clubfoot regarding the transverse talonavicular angle (*p* < 0.05) (24). We believed that the navicular bone still has a medial displacement despite the satisfactory appearance and functional activity of the clubfoot after the Ponseti method. The transverse talonavicular angle showed that the adducts deformity of clubfoot has not been completely corrected.

Ponseti noticed that the clubfoot had a strong tendency to relapse regardless of the approach used to obtain correction. Among the relapsed deformities, the most common is the recurrence of equinus deformity of ankle, followed by adducts deformity. The recurrence of clubfoot, according to Ponseti, was caused by non-compliance with braces, which might result in an abnormal relationship between the tarsal bones (3). In our study, even though the clinical correction and the motion of the foot and ankle are satisfactory, the talonavicular angle on transverse images of MRI showed statistical differences, suggesting that the adducts deformity may be incompletely corrected and therefore additional follow-up is required to rule out the possibility of adducts deformity recurrence. At the same time, the residual deformity is present in up to 20% of clubfoot treated by the Ponseti method (27). We speculated that the reason may be aberrant articular morphology. With MRI, we can detect these small variations in time so as to take targeted treatment and avoid residual abnormalities.

Several limitations of our study should be mentioned. First, long-term follow-up with a larger number of cases will be needed to exclude the possibility of recurrence of clubfoot. Second, the cost of MRI examination is too expensive and using them during the neonatal period is challenging because the infant must be sedated. At last, because the thickness of the MRI scan is 3–4 mm, it will cause errors in the measurement results when selecting slices.

## Conclusion

To our knowledge, although the appearance and function of clubfoot was recovered well after the Ponseti method, the transverse talonavicular angle still shows statistical differences on MRI. MRI can help us to better characterize clubfoot deformity and objectively assess the effectiveness of the Ponseti method. It may reveal recurrence and complications of clubfoot earlier than x-ray. The results of MRI showed that the Ponseti method successfully corrected cavus, varus, and equinus deformities and incompletely corrected the adduction deformity of clubfoot. Ponseti method may cause dorsal talonavicular subluxation in the sagittal plane and lateral dislocation of the navicular bone in the transverse plane on MRI.

## Data Availability Statement

The original contributions presented in this study are included in the article/supplementary material, further inquiries can be directed to the corresponding author.

## Ethics Statement

This study was approved by life Ethics Committee of the Capital Institute of Pediatrics (No.SHERLLM2022015). Written informed consent to participate in this study was provided by the participants’ legal guardian/next of kin. Written informed consent was obtained from the individual(s), and minor(s)’ legal guardian/next of kin, for the publication of any potentially identifiable images or data included in this article.

## Author Contributions

ZL and JZ designed the study. JZ collected the database and images and wrote the manuscript. HL and NW measured the six major parameters and performed the statistical analysis. ZL, NW, and HL reviewed and revised the manuscript. All authors read and approved the final manuscript.

## Conflict of Interest

The authors declare that the research was conducted in the absence of any commercial or financial relationships that could be construed as a potential conflict of interest.

## Publisher’s Note

All claims expressed in this article are solely those of the authors and do not necessarily represent those of their affiliated organizations, or those of the publisher, the editors and the reviewers. Any product that may be evaluated in this article, or claim that may be made by its manufacturer, is not guaranteed or endorsed by the publisher.
